# Lipidomics and Transcriptomics Revealed Dietary Complex Plant Extracts Improve Lipid Composition of *Longissimus dorsi Muscle* in Sheep

**DOI:** 10.3390/foods14040688

**Published:** 2025-02-17

**Authors:** Hui Guo, Ruixue Nie, Wenwen Wang, Tao Guo, Chang Gao, Jinju Mao, Yuchao Hu, Na Liu, Xiaoping An, Yang Jia, Jingwei Qi, Yuan Wang

**Affiliations:** 1 College of Animal Science, Inner Mongolia Agricultural University, Hohhot 010018, China; guohui616@126.com (H.G.); nie@imau.edu.cn (R.N.); wangwenwen2017@emails.imau.edu.cn (W.W.); guot199608@163.com (T.G.); gaochang117@163.com (C.G.); m18353340167@126.com (J.M.); yuchaohu1994@163.com (Y.H.); liuna_dky@163.com (N.L.); xiaoping_an@163.com (X.A.); jiayang@imau.edu.cn (Y.J.); qijingwei@imau.edu.cn (J.Q.); 2 Key Laboratory of Smart Animal Husbandry, at Universities of Inner Mongolia Autonomous Region, Hohhot 010018, China; 3 Inner Mongolia Herbivorous Livestock Feed Engineering Technology Research Center, Hohhot 010018, China

**Keywords:** complex plant extracts, intramuscular fat deposit, lipid metabolism, meat quality, multi-omics

## Abstract

Dietary regulation of intramuscular fat (IMF) deposition and fatty acid composition offers an effective strategy to enhance meat nutritional value. As phytogenic supplements rich in bioactive compounds, complex plant extracts (CPE) have demonstrated potential in improving meat quality through lipid metabolism modulation while ensuring food safety. In this study, we used 36 female sheep, approximately 4 months old and with a similar weight (29.92 ± 2.52 kg), to investigate the effects of CPE supplementation (80 mg/kg) on lipid metabolism. After the 75-day standardized feeding trial, the sheep were subjected to humane slaughter procedures and collected the *Longissimus dorsi muscle* (LDM) for further experimental process. The findings indicate that CPE significantly increased (*p* < 0.05) the IMF content (36%) and total fatty acids concentration (10,045.79 to 26,451.99 ug/g). Lipid metabolism in LDM was mainly affected by regulating phospholipids (six lipid subclasses were affected). The qRT-PCR analysis showed that differential expressed genes, *PLA2G2D* and *PLA2G4E*, associated with lipid metabolism were significantly reduced. CPE appears to modulate the fatty acids through sphingolipid, linolenic acid metabolism, and glycosphingolipid biosynthesis pathways. Thus, this study uncoded the mechanisms of CPE on fatty acid, providing critical evidence that CPE can regulate the meat quality of ruminants.

## 1. Introduction

To balance the ecological environment, improve mutton production, and respond to the policy of banning and prohibited grazing policy, the raising mode of mutton sheep has been changed from the traditional grazing mode to the large-scale, intensive, and standardized house feeding [1]. This shift in the conditions of house feeding has changed the pattern of lipid metabolism (the enzyme activities of fatty acid synthase [FASN] and hormone-sensitive triglyceride lipase [HSL] were significantly lower), fat deposition (intramuscular fat [IMF] content increased by 24%), fatty acid composition (saturated fatty acid [SFA] increased 4%), and the nutritional and sensory qualities (cooking loss increased 16%) of mutton due to the lack of fresh forage intake and exercise [2].

Intramuscular fat (IMF) represents a critical component in meat science due to its abundance of phospholipids, including phosphatidylcholine (23.56%), phosphatidylethanolamine (15.97%), and phosphatidylinositol (7.33%) [3]. This lipid fraction serves as a key determinant of meat product quality through multidimensional impacts on organoleptic characteristics (juiciness, flavor profile, textural tenderness) and nutritional attributes associated with fatty acid composition [4,5]. Study has shown that plant extracts can regulate IMF content (increased 4%) and fatty acid profile (such as SFA from 54.4 to 53.3) in the mutton [6]. Plant polyphenols play a regulatory role in fatty acid metabolism by promoting fatty acid oxidation and inhibiting fatty acid synthesis in vivo [7,8]. Supplementing diets with plant extracts has shown improvements in meat quality by altering fatty acids profiles (total SFA from 43.99 to 41.14) [9] and affecting gene expression related to lipid metabolism (*FASN* increased by 43%, inhibited peroxisome proliferator-activated receptor γ [PPAR γ] expression by 26%) [10,11,12], which means adding additives to diets is important to improve meat quality.

Eugenol, cinnamaldehyde, and chili oleoresin are notable polyphenols known to change fatty acids composition. In vitro experiments have confirmed that eugenol can change the composition of fatty acids by affecting the biological hydrogenation process [13], and cinnamaldehyde can reduce the content of C18:0 and increase the content of C18:1n9c, C18:2n6c, and C18:3n3 [14]. Chili oleoresin, in particular, can modify fatty acid composition in the breast and leg muscles of broiler (the content of polyunsaturated fatty acids [PUFA] from 37.29 to 39.90 and from 39.1 to 41.65, respectively) [15]. Study also suggest that a combination of these three plant extracts improves fermentation characteristics (pH from 5.22 to 5.34, total volatile fatty acid [VFA] from 13.53 to 12.72), epithelial gene expression (the expression of *Caspase-8*, *Caspase-9*, *Caspase-3* genes and other three genes was significantly reduced), and bacterial communities (Bacteroidetes from 39.43% to 39.95%, Firmicutes from 20.76% to 29.85%, and Proteobacteria from 26.22% to 15.88%) of sheep [16]. Meanwhile, our previous study demonstrated that adding 80 mg/kg complex plant extracts (CPE) containing eugenol, cinnamaldehyde, and chili oleoresin to sheep’s diets can improve growth (average daily gain increased 19%) and slaughter performance (live weight before slaughter increased 1.7%), and the amino acid (phenylalanine increase 3%) and fatty acid composition (the content of C14:0, C15:0, C14:1, C16:1, and C17:1 significantly increased) of *Longissimus dorsi muscle* (LDM) [17]. In summary, studies have shown that eugenol, cinnamaldehyde, and chili oleoresin can improve fatty acid composition, while the impact of CPE on lipid deposition in sheep and its underlying mechanisms still require in-depth research.

Recently, lipidomics and transcriptomics have been increasingly applied as powerful and favorable methods to lipid metabolism research, providing valuable insights into the molecular mechanism behind phenotypic changes. Li et al. [3] utilized lipidomics to reveal that glycerophospholipids (GP) lipids are essential in regulating IMF deposition, underscoring the close connection between IMF content and lipid metabolism. At the same time, Xin et al. [18] investigated that Ningxia Tan sheep’s diets supplemented with Sophora alopecuroides utilizing transcriptomics and identified *ACSL3*, *PLIN2*, *ABCA1*, and *ANGPTL4* as candidate genes for improving sheep meat quality through regulating IMF deposition. These fundings indicate that lipidomics and transcriptomics can effectively elucidate the molecular mechanisms of IMF deposition and lipid metabolism.

Under house-feeding conditions, mutton lipid metabolism, fat deposition, and fatty acid profile are affected, often leading to an increase in SFA, which reduces its nutritional value [19]. Therefore, the objective of this study was to investigate the effects of CPE on the IMF content and fatty acid composition of LDM in sheep. The lipid composition, vital regulatory pathways, and candidate genes in LDM were analyzed to understand how dietary CPE supplementation influences lipid metabolism in sheep’s LDM.

## 2. Materials and Methods

### 2.1. Animal and Experimental Design

The complex plant extract (Pancosma, Rolle, Switzerland, XTRACT^®^ 7065) used in this study was provided by Pancosma, and the practical contents of eugenol, cinnamaldehyde, and capsicum oleoresin were 9.5%, 5.5%, and 3.5%, respectively. Animal tests were conducted at Inner Mongolia Agricultural University test base, Hohhot, Inner Mongolia, China. The ethics of the experimental protocol were approved by Inner Mongolia Agricultural University Research Ethics Committee (permission number [2020]065). The experiment lasted 75 days, including a 15-day pre-feeding phase and a 60-day formal study period. To eliminate the impact from sex, only female sheep were selected. Thirty-six sheep, approximately 4 months old and of similar weight (29.92 ± 2.52 kg), were randomly assigned to two groups of six pens each (six replicates per group), three animals per each pen.

The control group (CTRL Group) received a basal diet formulated according to the Meat sheep feeding standard (NY/T816-2004), while the experimental group (CPE Group) was fed the same basal diet supplemented with 80 mg/kg CPE. The details of the basal diet’s ingredients and nutrient levels are shown in Table A1. Animals were fed twice daily during the experimental period (08:00 a.m. and 05:00 p.m.) to ensure 10% refusal. Water was freely available throughout the experiment period.

On the 61st day of the trial period, one sheep per pen was randomly selected for slaughter. Sheep were fasted for 12 h, and water was prohibited for 2 h before slaughter. The slaughtering procedure followed Islamic guidelines, with blooding via the caudal vein. One sheep was randomly selected from per each pen for slaughter (a total of 12 sheep). After slaughter, 200 g of the LDM was collected from the sheep’s right penultimate ribs for determining IMF content, fatty acid composition, lipidomics, and transcriptomics.

### 2.2. Determination of IMF Content and Fatty Acid Composition

IMF content was measured using the FOSS automatic Soxhlet fat extraction system (ST 255) with Petroleum ether as the solvent, as described by Li J et al. [20]. For fatty acid analysis followed the method described by Guo Tao [17], tissue samples (55 mg) were pre-treated by weighing and homogenizing with 4 mL n-hexane, followed by shaking at 50 °C for 30 min. Then, 3 mL of a 0.4 mol/L KOH methanol solution was added and shaken again at 50 °C for 30 min. After adding another 1 mL of H_2_O and mixing well, the mixture was allowed to settle, and the upper layer was collected for injection of the further chromatographic analysis.

For chromatographic analysis, the treated samples were analyzed using an Agilent chromatographic system (GC-MS 7890B-5977A, Agilent, Santa Clara, CA, USA) by the GC-MS external standard method. The column used was a DB-23 (30 m × 320 μm × 0.25 μm). The temperatures of inlet and detector were 250 °C and 230 °C, respectively, with a shunt injection ratio of 1:5. Helium was used as the carrier gas, with a sample volume of 1 μL. The heating procedure of the column box was as follows: an initial temperature hold at 50 °C for 1 min, followed by a ramp to 175 °C at 25 °C/min, and then a further increase to 230 °C at 4 °C/min, where it was held for 24.75 min.

### 2.3. Lipidomic Analysis

Lipid metabolite extraction followed the method described by Matyash V et al. [21]. After extraction, UHPLC-MS/MS analyses were performed using a Vanquish UHPLC system (Thermo Fisher, Bremen, Germany) coupled with an Orbitrap Q ExactiveTM HF mass spectrometer (Thermo Fisher) in Novogene Co., Ltd. (Beijing, China). The LC-MS raw data were processed by using Compound Discoverer 3.01 (CD3.1, Thermo Fisher) to perform peak alignment and peak picking for each metabolite. The peak intensity was used for lipid quantification and was normalized to the total spectral intensity. After the peak intensities were normalized, the molecular formula based on additive ions, molecular ion peaks, and fragment ions was predicted. And then, peaks were matched with the Lipidmaps and Lipidblast database to obtain the accurate qualitative and relative quantitative data.

Lipid data results were then qualitatively and quantitatively analyzed, with data quality control applied before proceeding to bioinformatic analysis. The partial least squares discriminant analysis (PLS-DA) was performed at Python-3.5.0 to investigate group separation. The differentially altered lipid molecules (DALs) between the CTRL and CPE group were screened based on criteria, including VIP > 1.0, FC > 1.2, or FC < 0.833 and *p* < 0.05 (VIP refers to the projected importance of variables of the first principal component of the PLS-DA model, and *p*-value is calculated by *t*-test) [22]. Relative abundance of each lipid species was calculated as a percentage of the total lipid molecules. The correlations between individual DALs for each group were analyzed using Pearson correlation with the “SRplot”, an online platform for data analysis and visualization (Accessed on 9 November 2023, https://www.bioinformatics.com.cn).

### 2.4. Transcriptome Analysis

Following RNA extraction with Trizol (Ambion/Invitrogen, Waltham, MA, USA), RNA integrity and total amount were accurately detected using the Agilent 2100 bioanalyzer (Agilent). Library construction and quality control were performed after detection. Once libraries passed the test, they were pooled based on effective concentration and the target downstream data volume for Illumina sequencing, generating 150 bp paired-end reads. Raw reads were quality controlled to remove adapter sequences and other low-quality data processed through in-house perl scripts. Clean data were also calculated for Q20, Q30, and GC content. All downstream analyses were based on high-quality clean data. Clean reads were aligned to the reference genome, ARS-UI_Ramb_v3.0 using HISAT2, transcript abundance was calculated by using RSEM and normalized via the fragments per kilobase of exon model per million mapped reads (FPKM) method. Differential expression analysis between the comparison groups was performed using DESeq2 package (1.20.0) using thresholds: log2 (fold change) > 1, *p* < 0.05 [23]. Kyoto encyclopedia of genes and genomes pathway (KEGG) enrichment analysis was performed using R package “clusterProfiler” (3.8.1), with a significance threshold *p* < 0.05. Gene Set Enrichment Analyses (GSEA) were based on the local GSEA software (3.0.0). Integrative analysis for DALs and DEGs detectable by lipidomics and transcriptomics, Pearson statistical method was used to calculate the relative abundance within the CTRL and CPE group.

### 2.5. qRT-PCR Verification

Total RNA was extracted from the samples using an ultrapure RNA extraction kit (CW0581S, Kangwei Century Bioscience Co., Ltd., Beijing, China). RNA concentration and purity were measured with a NanoDrop^®^ ND-2000 spectrophotometer. PCR reactions were performed on a fluorescent quantitative PCR instrument (ABI 7500, Applied Biosystems, Waltham, MA, USA), with ACTB used as a housekeeping gene. The mRNA sequences of sheep phospholipase A2 group IID (*PLA2G2D*) and phospholipase A2 Group IVE (*PLA2G4E*) were retrieved from the official National Center for Biotechnology Information (nih.gov). Primers were designed using Primer premier 5.0 software, and the primer sequences (Table 1) were synthesized by Sangon Biotech Co., Ltd. (Shanghai, China). The relative gene expression levels of *PLA2G2D* and *PLA2G4E* was calculated using the 2^−ΔΔ CT^ method [24].

### 2.6. Statistical Analysis and Data Visualization

Results of each group were expressed as mean ± standard deviation (SD) with six replicates. Data analyses were performed using Statistical Analysis Software (SAS version 9.4; Cary, NC, USA) and R Software (version of R-3.4.3). Student’s *t*-tests were conducted to compare the two groups. The *p* < 0.05 indicates a significant difference, while 0.05 ≤ *p* < 0.10 means a variation trend. Graphical abstract was generated using Figdraw (www.figdraw.com). Volcano plots were performed with the R package ggplot2. Clustered heat maps were plotted with the R package pheatmap (1. 20. 0).

## 3. Results

### 3.1. Fatty Acid Composition

**Figure 1:**
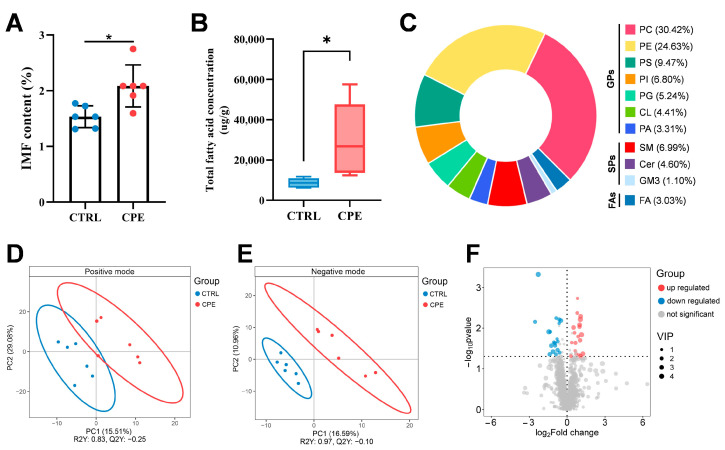
Effect of composite plant extracts (CPE) supplementation on lipid characteristics of the *Longissimus dorsi muscle* (LDM) in sheep (n = 6). (**A**) Intramuscular fat (IMF), expressed as means ± standard deviation. (**B**) Total fatty acid concentration, box plots with median (inside horizontal line), 25th and 75th percentiles (lower and upper edges of the boxes, respectively), minimum and maximum (lower and upper lines outside the boxes) values. (**C**) Percentage of lipid subclasses for pooled two groups. (**D**) Partial least squares discriminant analysis (PLS-DA) score spots in positive iron mode. (**E**) PLS-DA score spots in negative iron mode. (**F**) The volcano plot showed all lipid molecules detected from the CTRL and CPE groups, with differentially upregulated and downregulated lipid molecules highlighted in red and blue, respectively. * *p* < 0.05. CTRL, fed a basal diet. CPE, fed the basal diet supplemented with CPE. GPs: glycerophospholipids. SPs: sphingolipids. FAs: fatty acyls. PC: phosphatidylcholines. PE: phosphatidylethanolamines. PS: phosphatidylserines. PI: phosphatidylinositol. PG: phosphatidylglycerol. CL: cardiolipins. PA: phosphatidic acids. SM: sphingomyelin. Cer: ceramide. GM3: acidic glycosphingolipids. FA: fatty acids and conjugates.

As shown in Figure 1A and Table A2, supplementation with CPE significantly increased the IMF content (36%) in the LDM of sheep (*p* < 0.05). Additionally, the total fatty acid concentration (from 10,045.79 reduced to 26,451.99 ug/g) in LDM was also measured, revealing that Sheep in the CPE group exhibited a higher fatty acid concentration than those in the CTRL group (Figure 1B and Table A2). The fatty acid composition analysis, detailed in Table 2, identified a total of 26 fatty acids. Specifically, CPE supplementation significantly reduced the levels of C6:0, C11:0, C18:0, C18:3n6, and C22:0 (*p* < 0.05), while C8:0 showed a slight downward trend (*p* = 0.079). Moreover, sheep in the CPE group had elevated concentrations of C14:1 and C18:3n3 (*p* < 0.05), with the concentration of C17:1 showing a slight increase (*p* = 0.089). Compared to the CTRL group, supplementation CPE did not significantly impact the levels of SFA, unsaturated fatty acids (UFA), PUFA, and monounsaturated fatty acids (MUFA) (*p* > 0.05, Table 3).

### 3.2. Lipid Profile

To identify the lipid molecules in LDM, lipidomic analysis was conducted using an UHPLC system in both positive and negative polarity modes. Lipid molecules were identified by mass-to-charge ratio (m/z), retention time, and a high match accuracy with the lipid library entries. Based on these criteria, 1088 lipid molecules were detected in the LDM of the CTRL and CPE groups. These lipid molecules were mainly categorized into GPs, sphingolipids (SPs), and fatty acyls (FAs), consisting of 917 GPs (84.28%), 138 SPs (12.68%), and 30 FAs (3.03%) (Figure 1C). GPs exhibited the greatest diversity, with phosphatidylcholine (PC) and phosphatidylethanolamine (PE) being the most abundant (Figure 1C). The results from partial least squares discriminant analysis (PLS-DA) showed a clear separation between CTRL and CPE groups, with R2Y = 0.83, Q2Y = −0.25 in the positive mode (Figure 1D), and R2Y = 0.97, Q2Y = −0.10 in the negative mode (Figure 1E).

**Figure 2:**
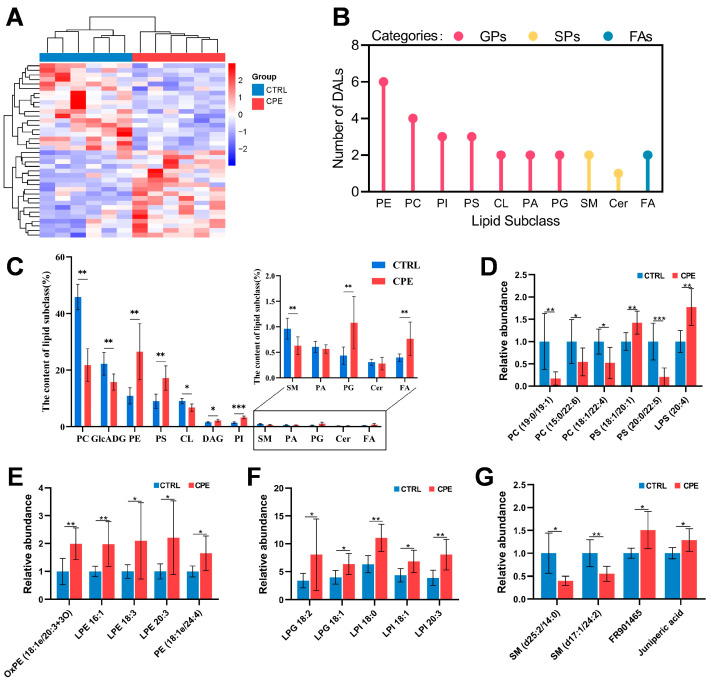
Comparison of lipid profiles in *Longissimus dorsi muscle* (LDM) between control (CTRL) and composite plant extracts (CPE) groups in sheep. (**A**) The heat map of the differentially altered lipid molecules (DALs) impacted by CPE, with blue for decreased and red for increased lipid levels. (**B**) The categorization of DALs. (**C**) Histogram of the proportion of lipid molecules between the CTRL group and the CPE group. Each column represents the percentage of that lipid molecule to all lipid molecules. (**D**–**G**) The relative content of phosphatidylcholines (PC) and phosphatidylserine (PS) (**D**), phosphatidylethanolamines (PE) (**E**), phosphatidylglycerols (PG), and phosphatidylinositol (PI) (**F**), sphingolipids (SP) (**G**). The mean value of each lipid molecule in the CTRL group was used to normalize the relative expression. Values are presented as means ± standard deviation (n = 6), * *p* < 0.05, ** *p* < 0.01, *** *p* < 0.001. CTRL, fed a basal diet. CPE, fed a diet supplemented with CPE added to the basal diet.

To further examine the effects of CPE on the lipid profile of LDM, DALs were identified according to the criteria mentioned previously. A comparison between the CTRL and CPE groups revealed 38 DALs (Figure 1F), with the CPE group exhibiting 19 upregulated and 19 downregulated DALs, as illustrated in Figure 2A. Of these 38 DALs, 27 lipid molecules were classified as GPs, SPs, and FAs, specifically with 22 as GPs, three as SPs, and one as FAs, while 11 remained unclassified (Figure 2B). Within the DALs, the relative contents of PCs and GlcADG were observed notably lower in the CPE group compared to the CTRL group (Figure 2C,D). Conversely, PEs, PSs, and PIs levels displayed higher in the CPE group than the CTRL group (Figure 2C,F). Furthermore, our findings revealed significantly low relative contents of SM (d25:2/14:0) and SM (d17:1/24:2), which belong to the SPs category, in the LDM of the CTRL group (Figure 2G).

### 3.3. Transcriptional Characteristics

**Figure 3:**
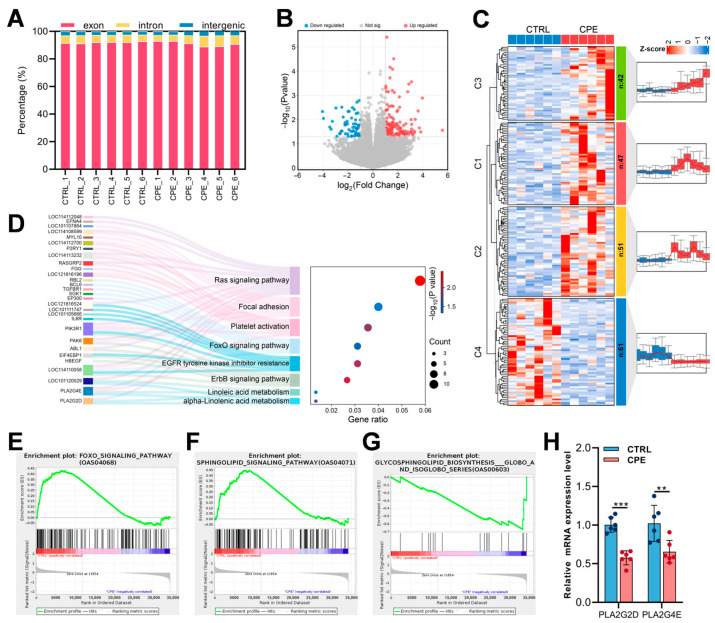
*Longissimus dorsi muscle* (LDM) tissue transcriptomic profiles of the control (CTRL) and composite plant extracts (CPE) groups. (**A**) Statistical diagram for the distribution of reads mapped to the reference genome. (**B**) Significantly up- and downregulated genes are represented as “red” and “blue” points in the volcano plot, respectively. (**C**) Heat map of four clusters in the comparison of CTRL vs. CPE. (**D**) KEGG enrichment analysis of DEGs between the CTRL and CPE groups. (**E**–**G**) GSEA enrichment result plots of CTRL vs. CPE comparison group. (**H**) Histogram of relative gene expression in LDMs. Values are presented as means ± standard deviation (n = 6), ** *p* < 0.01, *** *p* < 0.001.

To explore the gene expression profile of LDMs after CPE supplementation, RNA sequencing was performed on LDM samples from both CTRL and CPE groups (n = 6). This generated 82.03 Gb of raw data across 12 libraries, with at least 92.07% of the reads achieving quality scores of Q30 or higher (Table 4). On average, 90.44% of clean reads uniquely mapped to the reference genome (Table A2). In the annotation files for sequences that aligned uniquely, 91.53%, 5.73%, and 2.74% of reads aligned to exon, intron, and intergenic regions, respectively, per sample (Figure 3A). A total of 201 differentially expressed genes (DEGs) were identified, with 140 upregulated and 61 downregulated genes in the CPE group (Figure 3B). Cluster analysis revealed four gene clusters with distinct expression patterns between CTRL and CPE groups (Figure 3C). Specifically, DEGs in C1 (n = 47), C2 (n = 51), and C3 (n = 42) exhibited elevated expressions in the CPE group, while DEGs in C4 (n = 61) tended to be more highly expressed in the CTRL group.

As shown in Figure 3D, KEGG pathway enrichment analysis indicated that linoleic acid metabolism (oas00591) and α-linolenic acid metabolism pathways (oas00592) were directly related to lipid metabolism in LDM after CPE supplementation. Additionally, altered genes were also significantly enriched in pathways, such as Ras signaling, Focal adhesion, platelet activation, and FoxO signaling (Figure 3D). To further investigate new and key biological pathways potentially affected by CPE supplementation, GSEA of all genes expressed in CTRL and CPE groups was performed. Three pathways were of particular interest (Figure 3E–G), including the Foxo signaling pathway (oas04068), sphingolipid signaling pathway (oas04071), and glycosphingolipid biosynthesis globo and isoglobo series (oas00603).

Notably, *PLA2G2D* and *PLA2G4E* were enriched in both linoleic acid and α-linolenic acid metabolic pathways. Therefore, the expressions of these two genes were confirmed through qRT-PCR, which showed expression trends consistent with the transcriptome data (Figure 3H) (The expressions of *PLA2G2D* and *PLA2G4E* were reduced 42% and 33%, respectively).

### 3.4. Integrative Analysis of the Lipidomics and Transcriptomics

**Figure 4:**
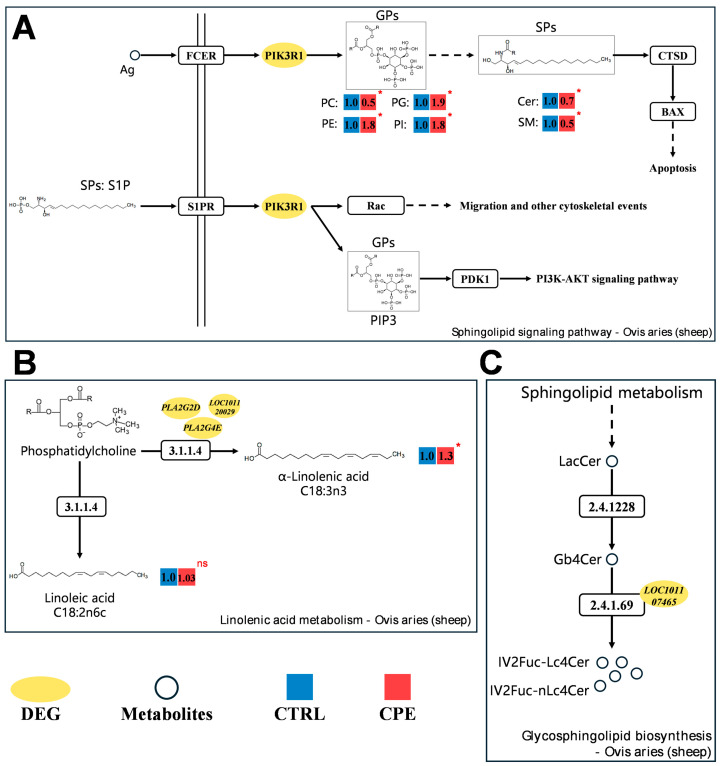
Proposed regulatory network involving relevant metabolic pathways in *Longissimus dorsi muscle* (LDM) after composite plant extracts (CPE) addition. (**A**) Sphingolipid signaling pathway. (**B**) Linolenic acid metabolism. (**C**) Glycosphingolipid biosynthesis. The yellow ovals indicate DEGs. Blue and red boxes represent CTRL and CPE groups, respectively, and the numbers above indicate the relative expression of lipid molecules. * *p* < 0.05, ns indicates no significant difference.

Next, we constructed three regulatory networks based on current findings, to explore the underlying mechanism of lipid metabolism alterations after CPE supplementation. There networks include the Sphingolipid signaling pathway, Linolenic acid metabolism, and Glycosphingolipid biosynthesis (Figure 4). The *PIK3R1* was enriched within the Sphingolipid signaling pathway, and significant changes were observed in various lipid molecules, such as PC, PG, PE, and so on (Figure 4A). This suggests that CPE supplementation may modulate multiple lipid molecules levels by regulating *PIK3R1* expression, which could in turn impact biological pathways, such as apoptosis, PI3K-AKT pathways, and Rac pathways, though these mechanisms require further investigation.

Linoleic acid is of great significance to human health. The findings indicated that DEGs and DALs were also distributed in the Linolenic acid metabolism signaling pathway (Figure 4B). Key genes like *PLA2G2D*, *PLA2G4E*, and *LOC10112009* showed significant expression changes, and fatty acid assays showed a significant increase in α-linoleic acid content. These findings implied that CPE may affect the synthesis of both linoleic and a-linoleic acid by altering the levels of key genes in this pathway.

Lipidomic analysis revealed that lipids from the SPs family are important components of IMF, and enrichment analysis in this part revealed that the glycosphingolipid biosynthesis pathway was enriched (Figure 4C). This pathway, initiated by sphingolipid pathway products, plays a key role in synthesizing lipid molecules of SPs. In more details, ceramides (Cer), sphingomyelin (SM), etc. in the SPs family were significantly altered, and *LOC101107465* gene expression was significantly increased after CPE supplementation. Therefore, we hypothesized that *LOC101107465* may affect SPs synthesis via this pathway, which in turn alters lipids composition within IMF.

## 4. Discussion

Numerous studies [6,20,21,25] have aimed to improve mutton’s nutritional quality, with plant extracts widely recognized as a promising, eco-friendly feed additive. Previously, we already demonstrated that adding 80 mg/kg of CPE to the sheep’s diets improved meat quality by regulating the fatty acids’ composition of LDM [17]. In this study, we supplemented the diet with 80 mg/kg of CPE and found that CPE effectively modulated lipid metabolism, increasing IMF content and fatty acid concentration, and changing fatty acid composition. Furthermore, by applying integrative lipidomic and transcriptomic analysis, we systemically evaluated the specific effects of CPE on the LDM lipid metabolic spectrum, and the expression profile of genes implicated in lipid metabolism. Based on these findings, we demonstrated that CPE modulates lipid metabolism and fatty acid profiles through the coordinated regulation of gene expression, lipid molecular composition, and metabolism-related pathways. Thus, we boldly hypothesize that CPE could improve the sensory qualities of lamb meat through the aforementioned pathways. Of course, further in-depth research is still needed to confirm this hypothesis.

IMF content is related to mutton’s flavor, tenderness, and juiciness. IMF, often referred to as marbling, is deposited within the membrane of muscle connective tissue, contributing valuable fatty acids and enhancing mutton’s nutritional value [26]. In this experiment, CPE could modulate the deposition of IMF by changing several signaling pathways and regulatory genes. Consistent with the previous study, the addition of CPE could improve meat’s sensory [17]. The functional fatty acids in mutton are primarily UFA [19]. Research indicated the phenolic active substances in plant extracts can affect the activities of Δ-5 desaturase and Δ-6 desaturase during PUFA synthesis, thereby increasing the PUFA levels, as observed in porcine psoas [27]. Consistent with this study’s findings, the addition of CPE with phenolic compounds notably increased α-Linolenic acid and PUFA by 3.64%, while eicosapentaenoic acid and docosahexaenoic acid levels remained unaffected in this study. Excessive intake of SFA is associated with an increased risk of obesity, insulin resistance, and cardiovascular diseases [28]. SFA can elevate serum low-density lipoprotein cholesterol (LDL-C) levels, leading to cholesterol deposition in the inner wall of an artery and increase the risk of cardiovascular diseases [29]. Meanwhile, MUFA have biological effects, including lowering blood sugar and cholesterol and protecting the heart, with their levels positively correlated with the sensory quality of meat [30]. In this study, the CPE group showed a reduction in the levels of C6:0, C11:0, C18:0, and C20:0 in the LDM of sheep, with the total SFA content decreasing by 3.54%. Additionally, C14:1 and C18:3n3 levels significantly increased, and C17:1 and UFA levels showed an upward trend. Our findings indicated that CPE regulates the lipid metabolism through detailed fatty acid composition, thus laying a solid foundation for further research.

Lipidomics analysis revealed that the supplementation of CPE in sheep’s diets affected lipid metabolism mainly by regulating the content of GP, such as PE, PI, PC, PG, CL, and PA. The GP plays a crucial role in lipid, lipoprotein, and energy metabolism, which was related to health and disease [26]. Our study showed that dietary CPE supplementation notably increased the IMF content in LDM, which is rich in phospholipids, like PC and PE [5]. PE, in particular, contains abundant UFA [31]. This study demonstrated that dietary supplementation of CPE significantly raised the PE content in the LDM, with a trend toward higher UFA levels. This suggests that CPE might enhance UFA content and improve the fatty acid profile of LDM by increasing PE levels. PG, primarily found in pulmonary surfactants and certain microorganisms, is a precursor to cardiolipin biosynthesis and is essential for lipid-protein and lipid-lipid interactions [26]. For instance, PG can inhibit PC transfer [32], and inhibition of PC transfer increased after significant upregulation of the PG lipid subclass, resulting in significant downregulation of PC. PC, a major phospholipid in eukaryotic cell membranes, is the largest phospholipid in mammalian cells and organelles, accounting for 45% to 55% of the total phospholipids in cells [26]. Dietary nutrition can affect PC distribution and levels by mediating fatty acid metabolism in muscle [33,34]. In this study, dietary CPE supplementation resulted in a significant downregulation effect on PC levels in LDM. This is consistent with the findings from Jiao et al. [35], where *Agriophyllum squarrosum* supplementation similarly downregulated PC content and modulated fatty acids in subcutaneous adipose tissue of Tan sheep.

PA, an important intermediate in lipid metabolism, serves as a precursor for both triglycerides and GP [36]. PI, another key phospholipid in animals, is a critical cell membrane component and a precursor to inositol phosphates—signaling lipid essential for growth and metabolism [37]. PI provides a source of essential lipids, including all phosphoinositol and sphingolipids containing inositol, DAG, and PA [37]. At present, although PI research in ruminants is limited, this study found that CPE supplementation significantly upregulated PI content and furthermore, increased DAG content, which required PI synthesis. This indicated that PI and DAG may serve as potential DALs of regulating lipid metabolism. The highlight of this research lies in using lipidomics to precisely clarify how CPE in sheep diets regulates lipid deposition, offering new insights into improving meat quality.

The molecular mechanism driving phenotypic changes can be revealed by transcriptomics. In this experiment, 201 DEGs were identified, with several related to lipid metabolism, such as *PLA2G2D* and *PLA2G4E*. The qRT-PCR analysis confirmed that relative expression levels for *PLA2G2D* and *PLA2G4E* genes were consistent with the transcriptomic data. *PLA2G2D* and *PLA2G4E* genes belong to the phospholipase A2 (*PLA2*) family, the enzyme that widely presents in mammalian tissues and plays a crucial role in phospholipid metabolism. *PLA2* is an essential metabolic and regulatory enzyme involved in physiologic intracellular and extracellular signaling, with roles in physiological and pathological reactions of various inflammation-related diseases [38]. Its physiological functions include cell signal transmission, lipid mediators’ production, phospholipid structure modification, and promotion of alveolar surface-active substances metabolism [39]. *PLA2* catalyzes the hydrolysis of UFA in GP [40]. In this study, dietary supplementation of CPE reduced *PLA2G2D* and *PLA2G4E* gene expressions in LDM, resulting in increased UFA content. This suggests that the downregulation of these genes weakens the catalytic effect of UFA hydrolysis. This change in expression may indicate that these two genes influence the synthesis of UFA through other genes or negative feedback regulatory pathways. However, the specific mechanisms require verification in further research.

As shown in the linoleic acid metabolism pathway, PC acts as a precursor for linoleate synthesis, producing C18:2n6c via *PLA2G2D* and *PLA2G4E*, which is subsequently converted to C18:3n6. However, further investigation is still needed to understand why no significant change was not observed in the metabolic of C18:2n6. Study have shown that α-linolenic acid metabolism affects fatty acid metabolism in the mammary glands of dairy cows [41]. Similarly, our findings indicated that CPE supplementation significantly regulated the α-linoleic acid metabolic pathway in sheep. Although *PLA2G2D* and *PLA2G4E* gene expressions involved in C18:3n3 production pathways were significantly decreased, C18:3n3 content increased, possibly due to alternative production pathways. Lourenço et al. [13] found that plant extracts like eugenol and cinnamaldehyde increased C18:3n3 content by influencing the biohydrogenation process of fatty acids in fermenters. This suggests that CPE may have a similar effect in the rumen of sheep.

Phosphorylation also plays a vital role in lipid metabolism, influencing phosphorylation of many genes in this pathway. Acetyl-CoA carboxylase (*ACC*) encoded by *ACACA*, is involved in fat synthesis and catabolic activities through dephosphorylation and dephosphorylation [42]. Moreover, the phosphorylation of lipid droplet-coated protein (*PLIN1*) is crucial in fat metabolism of adipose tissue by regulating lipolysis and fat storage in adipocytes [43]. It precisely regulates lipolysis and fat storage within adipocytes, thereby playing a critical role in maintaining the overall lipid balance. In this study, the supplementation of CPE led to a remarkable improvement in the fatty acid profile of LDM. The KEGG analysis further revealed a significant enrichment in phosphorylation. Based on these results, we propose that future research could focus on depth functional validation experiments to directly verify the role of these DEGs in mediating the effects of CPE on lipid metabolism in LDM. Overall, CPE shows great potential as a natural dietary intervention, and further exploration of its mode of action could have reaching implications for the agricultural and food industries.

## 5. Conclusions

In conclusion, this work examined the impact of CPE on lipid metabolism in LDM, revealing that CPE supplementation improved fatty acid composition, increased IMF content, and altered glycerophospholipid content. Additionally, the observed changes in the expression levels of *PIK3R1*, *PLA2G2D*, *PLA2G4E*, and *LOC101107465* suggest CPE’s involvement in sphingolipid and linolenic acid metabolism signaling pathway, thereby facilitating the synthesis of lipid molecules. These findings offer valuable insights into the application of CPE to enhance mutton quality and improve fatty acid profiles in a targeted manner.

## Figures and Tables

**Table 1:** Primer sequence used for quantitative qRT-PCR.

qRT-PCR Target	Sequences of Primers (5′-3′)	Product Size/bp
*PLA2G2D*	F: CTGCTACTGCGGATTTGG	165
	R: TTGGTGGAACACTGGACG	
*PLA2G4E*	F: GTCCCTCCATTGCCTCCC	162
	R: ACCACCCTCCGTTTCTGC	
*ACTB*	F: CAGCAGATGTGGATCAGCAAGCAG	111
	R: TTGTCAAGAAAAAGGGTGTAACGCA	

**Table 2:** Effect of CPE supplementation on fatty acid composition (%) of *Longissimus dorsi muscle* in sheep (n = 6).

Items	Groups ^1^		SEM	*p*-Value
	CTRL	CPE		
C6:0	0.012 ^a^	0.003 ^b^	0.003	0.045
C8:0	0.012 ^x^	0.008 ^y^	0.002	0.079
C10:0	0.076	0.082	0.008	0.649
C11:0	0.003 ^a^	0.002 ^b^	0.000	0.008
C13:0	0.008	0.007	0.001	0.397
C14:0	1.496	1.818	0.215	0.315
C14:1	0.111 ^b^	0.145 ^a^	0.007	0.008
C15:0	0.214	0.240	0.024	0.467
C16:0	25.243	24.764	0.293	0.274
C16:1	1.1201	1.462	0.178	0.204
C17:0	0.943	1.042	0.063	0.291
C17:1	0.455 ^y^	0.596 ^x^	0.053	0.089
C18:0	16.450 ^a^	14.926 ^b^	0.424	0.029
C18:1n9c	44.988	45.679	0.844	0.576
C18:2n6c	6.525	6.738	0.616	0.812
C18:3n6	0.142 ^a^	0.089 ^b^	0.015	0.036
C18:3n3	0.246 ^b^	0.326 ^a^	0.018	0.011
C20:0	0.067	0.061	0.004	0.368
C20:1	0.079	0.083	0.005	0.583
C20:2	0.197	0.202	0.014	0.794
C20:3n6	0.156	0.124	0.015	0.166
C20:4n6	1.316	1.442	0.062	0.183
C20:5n3 (EPA)	0.103	0.101	0.019	0.952
C22:0	0.010 ^a^	0.007 ^b^	0.001	0.039
C22:2n6	0.012	0.010	0.002	0.309
C22:6n3 (DHA)	0.076	0.062	0.014	0.470

^1^ The control (CTRL) group was fed the basal diet. The composite plant extracts (CPE) group was fed the basal diet supplemented with 80 mg/kg CPE. The data with a and b lowercase letters are used to indicate the level of significance of differences (*p* < 0.05), with x and y lowercase letters indicating a changing trend (0.05 < *p* < 0.1).

**Table 3:** Effect of CPE on Saturated fatty acid (SFA), Unsaturated fatty acid (UFA), Polyunsaturated fatty acids (PUFA), and Monounsaturated fatty acids (MUFA) of *Longissimus dorsi muscle* in sheep (n = 6).

Items	Groups ^1^		SEM	*p*-Value
	CTRL	CPE		
SFA	44.535	42.959	0.659	0.122
UFA	55.526 ^y^	57.056 ^x^	0.592	0.098
PUFA	8.773	9.093	0.681	0.746
MUFA	46.753	47.964	0.977	0.401
PUFA/SFA	0.197	0.212	0.015	0.520
n-6 PUFA	8.151	8.402	0.674	0.797
n-3 PUFA	0.425	0.489	0.043	0.320
n-6/n-3PUFA	19.172	17.198	2.382	0.744

^1^ The control (CTRL) group was fed the basal diet. The composite plant extracts (CPE) group was fed the basal diet supplemented with 80 mg/kg CPE. The data with x and y lowercase letters are used to indicate a changing trend (0.05 < *p* < 0.1).

**Table 4:** Summary of sequencing quality data set.

Sample ^1^	Raw Reads	Raw Bases (G)	Clean Reads	Clean Bases (G)	Q20 ^2^ (%)	Q30 ^2^ (%)	GC Content (%)
CTRL_1	48, 225, 610	7.23	46, 023, 660	6.90	97.42	92.99	52.14
CTRL_2	42, 038, 964	6.31	39, 982, 964	6.00	97.39	92.90	52.38
CTRL_3	45, 173, 638	6.78	43, 744, 804	6.56	97.15	92.31	52.26
CTRL_4	45, 636, 690	6.85	44, 157, 812	6.62	97.32	92.76	52.35
CTRL_5	46, 562, 830	6.98	44, 734, 252	6.71	97.07	92.07	52.31
CTRL_6	47, 475, 516	7.12	45, 549, 028	6.83	97.37	92.79	52.51
CPE_1	41, 269, 346	6.19	39, 376, 762	5.91	97.20	92.48	52.76
CPE_2	45, 220, 064	6.78	43, 131, 022	6.47	97.24	92.57	52.63
CPE_3	46, 173, 940	6.93	43, 625, 064	6.54	97.41	93.00	52.43
CPE_4	50, 098, 782	7.51	48, 478, 422	7.27	97.32	92.73	51.54
CPE_5	43, 483, 562	6.52	42, 194, 454	6.33	97.28	92.62	51.18
CPE_6	45, 541, 352	6.83	43, 554, 326	6.53	97.40	92.92	51.88

^1^ 12 samples from both control (CTRL) and composite plant extracts (CPE) groups. ^2^ represents the proportion of one incorrectly identified base per 1000 bases, with a correct recognition rate of 99.9%.

## Data Availability

The data presented in this study are openly available in the Sequence Read Archive (SRA)of NCBI, reference number [PRJNA1191029].

## Abbreviations

The following abbreviations are used in this manuscript:

IMF	intramuscular fat
PUFA	polyunsaturated fatty acid
CPE	complex plant extracts
GP	glycerophospholipids
PLS-DA	The partial least squares discriminant analysis
DALs	differentially altered lipid molecules
DEGs	differentially expressed genes
FPKM	fragments per kilobase of exon model per million mapped reads
KEGG	Kyoto encyclopedia of genes and genomes pathway
GSEA	Gene Set Enrichment Analyses
PLA2G2D	phospholipase A2 group IID
PLA2G4E	phospholipase A2 Group IVE
SD	standard deviation
SFA	saturated fatty acids
UFA	unsaturated fatty acids
MUFA	monounsaturated fatty acids
SPs	sphingolipids
FAs	fatty acyls
PC	phosphatidylcholine
PE	phosphatidylethanolamine
Cer	ceramides
SM	sphingomyelin
qRT-PCR	Quantitative polymerase chain reaction
PLA2	Phospholipase A2
ACC	Acetyl-CoA carboxylase
PLIN1	phosphorylation of lipid droplet-coated protein

## Appendix A

**Table A1:** Ingredients and chemical composition of the basal diet fed to the lambs ^1^.

Items	Content, %
Ingredients	
Maize grain	35.10
Soybean meal (43% crude protein)	5.00
Cottonseed meal	5.00
Corn germ meal	23.00
Sunflower seed shells	13.00
Rice bran meal	12.00
Limestone	1.50
Salt	0.70
Vitamin-mineral premix1	2.00
Dicalcium phosphate	0.70
Bentonite	2.00
Total	100.00
Chemical composition, %	
Metabolizable energy, MJ/kg ^2^	9.36
Crude protein, %	14.39
Neutral detergent fiber, %	33.99
Acid detergent fiber, %	12.97
Ash, %	10.01
Calcium, %	1.01
Phosphorous, %	0.69

^1^ The premix provided the following per kilogram: vitamin A 350000 IU, vitamin D3 93750 IU, vitamin E 938 mg, vitamin K3 63 mg, vitamin B1 62 mg, vitamin B2 188 mg, nicotinamide 750 mg, pantothenic acid 500 mg, vitamin B6 62 mg, biotin 3.7 mg, folic acid 38 mg, Se 18 mg, Zinc 3000 mg, iodine 23 mg, Co 30 mg, Mn 2500 mg, Fe 3240 mg, Cu 500 mg. ^2^ Metabolizable energy was calculated, and other nutrients were measured.

**Table A2:** Effect of composite plant extracts (CPE) supplementation on intramuscular fat (IMF) content and total fatty acid concentration of the LDM.

Items	Groups ^1^		SEM	*p*-Value
	CTRL	CPE		
IMF content (%)	1.53 ^b^	2.09 ^a^	0.28	0.010
Total fatty acid concentration (ug/g)	8625.08 ^b^	29,876.27 ^a^	45,660.25	0.034

^1^ The control (CTRL) group was fed the basal diet. The composite plant extracts (CPE) group was fed the basal diet supplemented with 80 mg/kg CPE. The data with a and b lowercase letters are used to indicate the level of significance of differences (*p* < 0.05).
